# JGES as a Platform for Knowledge Dissemination

**DOI:** 10.4103/0974-1216.71608

**Published:** 2009

**Authors:** Prakash Trivedi

**Affiliations:** National Institute of Laser and Endoscopic Surgery, 1,2,3, Gautam Building, Opp. Balaji Temple, Tilak Road, Ghatkopar (East), Mumbai - 400 077, India. E-mail: dr.ptrivedi@gmail.com

**Figure:**
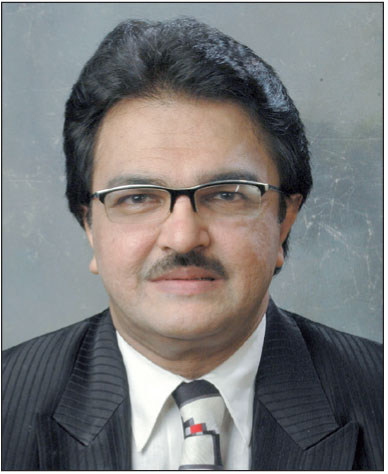


Dear Colleagues,

We stand today due to the untiring efforts of the founders of IAGE, the past Presidents, office bearers and members of the Managing Committee of IAGE over decades. The first volume of *Journal of Gynecological Endoscopic Surgeries* published was due to the responsibility given by Dr. H D Pai, IAGE immediate past President, to me to work hard as a Managing Editor with the support of others.

It is indeed an exceptional responsibility and pleasure to be the Managing Editor of the second volume of *Journal of Gynecological Endoscopic Surgeries* with a changing scenario of receiving articles from most experienced to amateur authors and from large centers to small places in India. There is one section of gynecological endoscopists, who, in spite of presenting their large amount of work in talks, fail to write articles of great benefit, may be because a skilled endoscopist does not necessarily mean that he/she is equipped with proper skills of journal article writing. Few others are not interested and few apparently have no time, all the excuses are correctible as there are persons good at writing articles and who can help them. The Indian endoscopist has to learn to rise to report, write, and share, even if it means to take help from the juniors or seniors, for a global impact of *Journal of Gynecological Endoscopic Surgeries* with more contributors.

We should remember that the busiest consultant and the one with greatest skills in the world always has time to share their experience with thousands of IAGE members.

This second volume has a good blend of endoscopic surgical excellences of laparoscopic myomectomy, scientific comparative evaluation of urinary incontinence correction, cost-effective slings, hysteroscopy in abnormal uterine bleeding, laparoscopic removal of ovarian cysts in rural set up, and excellent unusual cases. The advent of new energy sources for laparoscopic hysterectomy, along with its glamour and cost, can cause major ureteric injuries with experts and amateurs alike.

A special touch is given on the legal expectation of Care or Cure by an expert. A good journal has an eye into future not only with techniques/new gadgets but also with law.

The newly found bonding of IAGE office bearers and managing committee members makes us comfortable and confident that we will reach close to international standards, and I thank all for their continuous confidence in assigning the job of Managing Editor of *Journal of Gynecological Endoscopic Surgeries*.

